# Evidence of Low-Habitat Contamination Using Feathers of Three Heron Species as a Biomonitor of Inorganic Elemental Pollution

**DOI:** 10.3390/ijerph17217776

**Published:** 2020-10-23

**Authors:** Luca Canova, Michela Sturini, Antonella Profumo, Federica Maraschi

**Affiliations:** Department of Chemistry, University of Pavia, I-27100 Pavia, Italy; michela.sturini@unipv.it (M.S.); antonella.profumo@unipv.it (A.P.); federica.maraschi@unipv.it (F.M.)

**Keywords:** Grey Heron *Ardea cinerea*, Little Egret *Egretta garzetta*, Cattle Egret *Bubulcus ibis*, biomonitoring of inorganic elements, trophic ecology

## Abstract

The concentration of 12 elements (As, Cd, Co, Cr, Cu, Fe, Hg, Mn, Ni, Pb, Se, and Zn) has been investigated in the feathers of three species of Ardeidae, namely the Grey Heron *Ardea cinerea*, the Little Egret *Egretta garzetta*, and the Cattle Egret *Bubulcus ibis,* all breeding at a colony located in the southern Padana Plain (NW Italy). This study is a first step for an evaluation of possible direct effects of these elements on chicks’ survival and growth rate. Fe, Zn, Cu, and Mn were in the range 7–69 mg Kg^−1^, while lower levels of Pb, Ni, As, and Se (0.27–1.45 mg Kg^−1^) were measured. Co, Cd, and Cr were close to the method detection limits (MDLs) in all the species. The measured concentrations of the most abundant trace elements, such as Zn and Cu, seem to reflect the geochemical pattern of the background (running water and soil), while Hg concentration is lower and it appears to be biomagnified, particularly in Grey Heron feathers. Its concentration is higher in adults than in chicks, and it differs among the three species, as it is closely related to the fish-based dietary pattern. The measured trace elements’ concentrations are below the threshold levels in all the heron species, and consequently, harmful and acute effects on the local population are unlikely; the conservation status of herons populations in northern Italy is probably more affected by other factors, such as climate changes, altered aquatic environment, and, consequently, food quality.

## 1. Introduction

Inorganic elemental pollutants enters the environment due to anthropogenic activities, such as industry, agriculture, and natural processes. They are present in rivers, lagoons, and marshes, which are currently the most threatened aquatic ecosystems in the world [1,2]; some elements persist in the environment for extended periods and, due to their high bioavailability in water [3], they can concentrate through the food chain and negatively affect birds foraging in the aquatic habitats [4,5].

Biological organisms, at different trophic levels, are often used as bioindicators to provide evidence of the potential adverse effects of exposure to contaminants [6,7]. Monitoring of metals in bird species is useful to assess their health and, at the same time, to evaluate the degree of contamination of the ecosystems where they live [8,9].

Exposure to high levels of metals is associated with various neurological, physiological, reproductive, developmental, and behavioral disorders of birds [10,11]. Moreover, it is responsible for oxidative damage, as detoxification and excretion are expensive metabolic processes leading to oxidative stress [12,13]. High concentrations of heavy elements can result in eggshell thinning, reproductive failure and immunosuppression, adverse developmental effects, as well as embryo malformations and mortality, which are all factors contributing to population decline. For instance, exposure to cadmium (Cd), mercury (Hg), and selenium (Se) negatively affects the body mass/condition and health of birds by reducing their growth or body weight and, consequently, it harms survival and reproductive success [14,15]. Hg accumulated in body tissues can adversely affect reproduction, especially in higher trophic level species, such as fish-eating birds [16,17,18].

Colonial herons are species of special concern in Europe; their conservation status has been considerably improved in the last 30 years and many heronries are currently protected. However, several species are threatened by factors that overrule local and European authorities, such as drought and habitat destruction in African wintering quartiers for migrating species, change in water and food availability and quality in Southern Europe, as well as pollution and reduction of suitable breeding sites in Italy. Water pollution can be an important threat both for adults and chicks, since nearly all the species feed on aquatic prey and can bioaccumulate and biomagnify harmful pollutants. 

Herons can be good indicators of elemental exposure for different reasons including the following: (i) they feed in the aquatic habitat; (ii) they are long-lived organisms; (iii) they are secondary consumers, and thus exposed to a wide range of chemicals; and (iv) their position at the top of the food chain makes them susceptible to bioaccumulation of heavy metals from various sources [19,20,21]. Many tissues of birds, such as feathers, liver, bones, and blood, but also eggs and eggshells, have been used to monitor avian exposure and assess the risk [22,23,24,25]. Among these, feathers have many advantages because they can be obtained quickly and repeatedly from the same individual without affecting its health, and their preservation does not require refrigeration [25]. Moreover, in the case of herons, feathers reflect the local contamination better than other tissues, because chicks feed on prey collected close to the colony, and considerable amounts of elements are stored in the feathers during the breeding of chicks [26].

In this work, we analyzed twelve elements, arsenic (As), cadmium (Cd), cobalt (Co), chromium (Cr), copper (Cu), iron (Fe), mercury (Hg), manganese (Mn), nickel (Ni), lead (Pb), selenium (Se) and zinc (Zn) in feathers of three heron species breeding in NW Italy. The main objectives of the research were to (i) estimate concentrations levels of trace elements in feathers of both adults and chicks, (ii) detect a possible risk for the conservation status in three heron species (Grey Heron *Ardea cinerea*, Little Egret *Egretta garzetta*, and Cattle Egret *Bubulcus ibis*), (iii) identify a possible relation among elements’ concentrations and diet of the examined species, and (iv) provide useful data for future comparisons and observations.

## 2. Materials and Methods

### 2.1. Sample Collection

We collected feathers of three breeding heron species, Little Egret *Egretta garzetta*, Grey Heron *Ardea cinerea*, and Cattle Egret *Bubulcus ibis* in the Sic IT2090001 “Monticchie” (NW Italy, coordinates 45°08′43.12′′ N, 9°39′26.34′′ E) in May 2018 and 2019, corresponding to the late breeding season for these species. Adults’ and chicks’ feathers, wing or chest, were collected inside nests or from the body and quickly preserved in plastic bags. In order to avoid data redundancy, a single feather was collected from each chick lying in each nest. Sixty-five feathers were collected from both young and adult herons as follows: *A. cinerea* (12 adults and 5 chicks), *E. garzetta* (15 adults and 8 chicks), and *B. ibis* (17 adults and 8 chicks).

### 2.2. Laboratory Analyses

Ultrapure HNO_3_, Trace-SELECT^®^ (65% w/w), H_2_O_2_ (30% w/w), and certified multi-standard solution Merck VI for ICP-MS were purchased from Sigma-Aldrich (Milan, Italy). The multi-element standard solutions were prepared daily in 0.5% ultrapure HNO_3_. Ultrapure water was produced in the laboratory by a Millipore Milli-Q system. 

A CEM Mars microwave oven (CEM s.r.l., Cologno al Serio, Italy) equipped with eight PTFE vessels (Xpress, 55 mL) was used for sample digestion. XpressVapTM accessory was employed for the evaporation of the digested acid solutions. Measurements were performed using an inductively coupled plasma quadrupole mass spectrometer (ICP-MS) (Elan DRC-e, PerkineElmer, Shelton, CT, USA) equipped with standard ICP torch, cross-flow nebulizer, nickel sampler, skimmer cones, and dynamic reaction cell™ (DRC). 

### 2.3. Analytical Procedure

The feathers were vigorously washed in deionized water, sonicated for 5 min in in acetone/H_2_O 1:10) and air-dried before the dissolution step. The whole feather from each individual was processed. Feathers, 20–150 mg, were accurately weighed into the PTFE vessels of the microwave digestion system, and 5 mL of HNO_3_ plus 2 mL of H_2_O_2_ were added. Then microwave heating was performed at 1600 W for 15 min, 200 ℃. After cooling, the contents were evaporated to a small volume (about 0.5 mL), diluted to 10 mL with MQ water in calibrated polypropylene tubes, and analyzed by DRC-ICP-MS for metals determination. Three-point calibration curves were generated in the range 5–500 µg L^−1^. Method detection and quantification limits (MDLs, MQLs) were obtained from the instrumental detection and quantification limits (IDLs, IQLs) calculated using the residual standard deviation (Sy/x) of the linear regression parameters as (3.3 × Sy/x)/slope and (10 × Sy/x)/slope, respectively, referred to in the overall procedure (See Table A1). Reagent blanks were prepared following the same procedure applied to samples. Five sets of method blank and certified reference material (BCR-397, trace elements in human hair) were processed and analyzed concurrently with the samples. The final concentrations were reported as mg/Kg dry weight.

### 2.4. Statistical Analyses

Univariate analysis of variance was used to test differences in the concentration levels of elements among species, years, and age, and a post hoc multiple comparison Tukey test was carried out to investigate differences among species when the null hypothesis in one-way ANOVA was rejected. Discriminant function analysis was run on log-transformed values of the significantly different variables, to obtain multivariate discrimination and a classification criterion. The non-parametric Mann–Whitney U test was adopted to test for differences in Se/Hg ratio between species and age classes. All the analyses were performed using the SPSS statistical software (IBM SPSS Statistics Inc. version 13.0). 

## 3. Results

Descriptive statistics of the investigated trace elements in the feathers of Grey Heron *Ardea cinerea*, Little Egret *Egretta garzetta*, and Cattle Egret *Bubulcus ibis* are listed in Table 1. Variance analysis was carried out to test the difference in elements’ concentrations among species and year.

Concentrations in feathers did not change among years (F_1,63_ = 0.27, *p* = 0.60), while significant differences occurred among species. In particular, relatively high levels of Zn (from 45 to 69 mg Kg^−1^ in Cattle Egret and Gray Heron, respectively), Fe (from 57 to 63 mg Kg^−1^ in Cattle Egret and Gray Heron, respectively), Cu (from 10.3 to 17 mg Kg^−1^ in Gray Heron and Cattle Egret, respectively) and Mn (from 7 to 13 mg Kg^−1^ in Little Egret and Grey Heron, respectively) were detected. Fe, Zn, Mn, and Cu concentrations decreased following the order Fe > Zn > Cu > Mn in Little Egret and Cattle Egret, while in Grey Heron the order was Zn > Fe > Mn > Cu. Hg and Ni levels were significantly higher in Grey Heron (2.8–1.3 mg Kg^−1^) than in Little Egret (0.7–0.57 mg Kg^−1^) and Cattle Egret (0.7–0.50 mg Kg^−1^, F_2,62_ = 57.5, *p* < 0.0001), while the contrary was observed for As (F_2,62_ = 4.47, *p* = 0.015). There were no significant differences in Pb and Cr levels among the species; Co and Cd were close to the MDLs for all species, and thus they were discarded from further analyses.

Post hoc comparisons showed differences between the Grey Heron and the Little Egret-Cattle Egret pair (Tukey HSD test, Table 1). Significantly higher concentrations of Zn, Hg, Mn, and Ni, and lower concentrations of Cu and As, were detected in Grey Heron and Little Egret, while Zn, Hg, Mn, Cr, Ni, and Se were lower in Cattle Egret than in Grey Heron. There were no significant differences in element concentrations between Little Egret and Cattle Egret (Table 1). 

**Figure 1:**
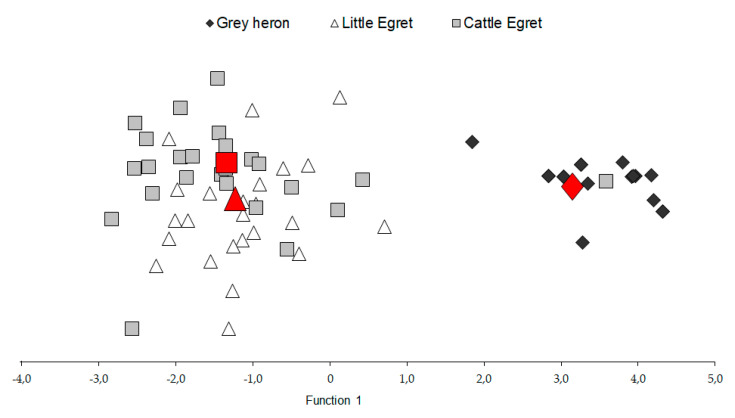
Discriminant analysis and group centroids (◆ Grey Heron; Δ Little Egret; ◼ Cattle Egret). Red geometric shapes show group centroids. Since function 1 accounts for more than 98% of total variances, only the distribution on the x-axis is discussed in the text.

A discriminant analysis was run on log-transformed concentration values that significantly differed in variance (Table 1). As shown in Figure 1, Grey Heron was well separated from Little Egret and Cattle Egret along a single axis, which accounted for 98.4% of the total variance (Wilk’s lambda = 0.16, χ^2^ = 106.5, *p* < 0.001); Hg and Zn showed the highest absolute correlation with a discriminant function that predicted 71% of the total cases correctly.

**Figure 2:**
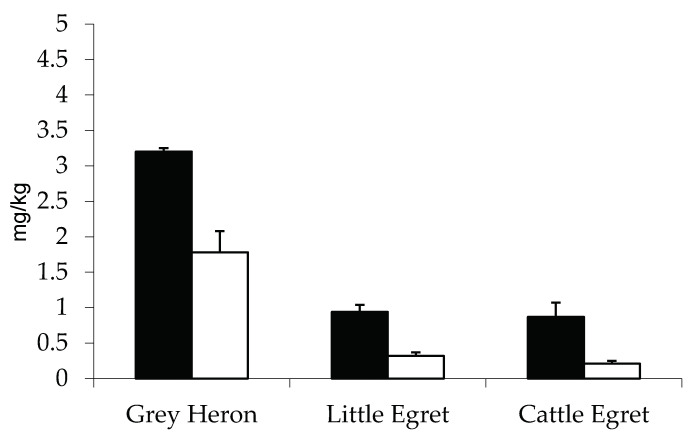
Average concentration and SE of mercury (Hg) in adults’ (black) and chicks’ (white) feathers. Data are expressed as mg Kg^−1^ ± SE.

Elements’ concentrations in adults and juveniles are listed in Table 2. Significant differences were observed for some of them, in particular for two metals in Cattle Egret, five metals in Little Egret, and six metals in the Grey Heron samples. Concentrations were significantly higher in adults than in chicks, as evident for As, Cu, Hg, and Mn but not for Fe and Se in Grey Heron; Hg but not Mn in Cattle Egret; and Cu, Hg, Pb, Cr, and Ni in Little Egret. Hg concentrations were significantly higher in adults than in chicks for all the species; the lowest differences occurred in Grey Heron and the highest occurred in Cattle Egret (Figure 2).

**Figure 3:**
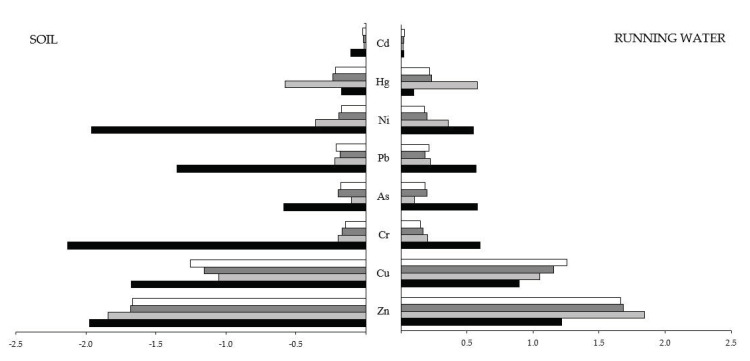
Comparison of the concentrations of the trace elements in the background (soil [27], water [28]) and in the feathers (black, background; pale grey, Grey Heron; dark grey, Little Egret; white, Cattle Egret). Data are log_10_ +1 transformed to emphasize the comparison.

Figure 3 shows an overview of the elements’ concentrations, in the logarithmic scale, in the background surrounding the study area (running water, µg/L and soil, mg/kg) for eight out of the 12 trace elements. Zn and Cu are the most abundant elements both in the background and feathers; Cr, As, Pb, and Ni levels are higher in the background than in feathers; Hg levels are higher in feathers than in background; Cd is the element with the lowest concentration in all samples.

## 4. Discussion

The study of the effects of heavy metals and elements on the ecology of vertebrates is useful to understand their impact on behavior, reproduction, survival [29,30] and, consequently, to improve conservation management for several species [31,32]. Many species of Ardeidae are gregarious, and the distribution of colonies is strongly aggregated; as a consequence, they share relatively restricted foraging areas and may be exposed to potential risks when foraging habitats are polluted [33]. 

The Lambro and Po rivers flow onto the Padana Plain (NW Italy) from North to South and from East to West respectively, the former collecting pollutants mainly from industrial activities and the latter from agriculture [34]; The Lambro river, which has been one of the most polluted rivers of Italy since World War II, flows into the Po river at less than 5 km from the site where the samples were collected, a radius that overcompasses the average foraging distance of several heron species. 

### 4.1. Fe, Zn, Cu, and Mn

As expected, the most abundant elements in Grey Herons’ feathers are Cu, Zn, Fe, and Mn. They are ubiquitous elements, considering their widespread distribution in the terrestrial and aquatic environments of the Mediterranean basin. The Zn concentrations range from 6 to 86 mg Kg^−1^ in Little Egret, from 9 to 64 mg Kg^−1^ in Cattle Egret, and from 50 to 83 mg Kg^−1^ in Grey Heron (Table 1). A toxicosis threshold limit of 1200 mg Kg^−1^ was reported by Solgi et al. [35]; therefore, detrimental effects on the population investigated in the present study can certainly be excluded. Copper concentrations vary from a minimum value of 7.1 mg Kg^−1^ in Grey Heron to a maximum of 80 mg Kg^−1^ in Cattle Egret, while the average concentration ranges from 10.3 in Grey Heron to 17 mg Kg^−1^ in Cattle Egret. 

The high concentrations of Cu and Zn, also present in the background, may be due to the extensive use of fungicides and bactericides in agriculture and to the presence of residue from electro-galvanic industrial activities in Northern Italy, especially in the metropolitan areas around the Lambro river [34]. Similar results (range 43–53 mg Kg^−1^) were reported by Rubio et al. [36] for Little Egret in the Odiel delta and by Eisler [37] in body tissues of birds feeding in polluted areas. In general, Mn, Zn, Fe, and Cu patterns are similar to those reported in the literature for the same species from different habitats, suggesting that these concentration levels represent both background and ecological levels [36,38].

### 4.2. As, Pb, Cr, Ni, Cd, and Co

Arsenic concentration ranges from 0.01 (all species) to 2.3 mg Kg^−1^ (Cattle Egret) and from average levels of 0.27 in Grey Heron to 0.6 mg Kg^−1^ in Little Egret, values that are comparable with those reported in the literature and far below the average concentration of 10 mg Kg^−1^ measured in feathers from contaminated sites [39]. The effects of As on ecosystems are poorly known, especially in birds. As reported by Sanchez-Virosta et al. [40], its presence in body tissues has influenced reproductive success and wing growth; it has induced apoptosis and autophagy in mucous membrane cells of birds gizzard [41], and it has behaved as an endocrine disruptor causing individuals death [42,43]. Birds that feed on arthropod and aquatic macroinvertebrates may accumulate As and levels of As in bird feathers reflect its presence in the background or preys at the upper trophic level, as suggested by Lucia et al. [44] and Ali and Khan [45]. Data reported in the present work were close to those observed in heron populations where no detrimental effects were observed [46]. Consequently, we may assume that As concentration, up to 2.3 mg Kg^−1^, should not harm the investigated population.

The environmental effects of Cr are well known [47]. Some of its chemical forms, primarily hexavalent chromium, are toxic. Cr levels higher than 2 mg Kg^−1^ in bird feathers can affect embryo development, hatching success, kidney damage [48], reduction of bone growth rate [19], and chick viability [49]. In the present study, the Cr average values were 0.48 in Little Egret, 0.40 in Cattle Egret, and 0.59 mg Kg^−1^ in Grey Heron, and they were close to those reported by Rubio et al. [36]. On the contrary, they were one order of magnitude lower than tissue concentrations measured by Eisler [47]. The Cr levels detected in the present study do not pose a severe threat to local bird populations.

Lead is a toxic metal to wildlife, especially in gamebirds such as Anatidae and Galliformes [50]. The use of lead shot in hunting can cause toxic or sublethal effects, altering hematocrit level, impairing breeding activity, and reducing clutch sizes as observed in seagulls [51], in Great tit *Parus major* [52], and [53] Common eider *Somateria mollissima*. Pb concentrations of 4 mg Kg^−1^ in feathers are known to be potentially toxic [16], and levels exceeding a threshold concentration have been related to lower eggshell thickness and impaired eggshell structure [54], decreased survival of nestlings, altered recognition of siblings, and thermoregulation imbalance [48]; even lower Pb concentrations can induce severe histopathological damages and necrosis associated with reduced enzymatic functionality [55]. 

In the present study, Pb levels range from 0.15 to 1.67 mg Kg^−1^ in Little Egret and Cattle Egret and the average concentrations vary from 0.53 and 0.67 mg Kg^−1^ in Little Egret and Grey Heron, respectively; all measured values are about one order of magnitude lower than the threshold limit.

Nickel roles in bird metabolism are complex, and birds probably adopt eggshells as excretion pathways more than feathers [56]. Ni concentrations in avian tissues have rarely exceeded 2.0 mg Kg^−1^ and only occasionally reached 5 mg Kg^−1^; higher values have been detected in herring gulls (9.9 mg Kg^−1^) and mallard (12.5 mg Kg^−1^) from heavily polluted areas [37]. It has been demonstrated that some species, such as duck chicks and adult, fed daily with high Ni concentration (up to 800 mg Kg^−1^) showed no or slight adverse effects, although high Ni levels (68 mg Kg^−1^) were detected in feathers after regrowth [37]. In the present study, Ni values range from 0.11 mg Kg^−1^ in Little Egret to 2.80 mg Kg^−1^ in Grey Heron, and from an average concentration of 0.50 in Cattle Egret to 1.3 mg Kg^−1^ in Grey Heron. These data were comparable with another study carried out on Heron species where no evidence of single nickel-mediated detrimental factors was observed [36]. 

### 4.3. Hg and Se

Natural and anthropogenic processes lead to the release of inorganic Hg (IHg) [57] that can be methylated in monomethylmercury (MeHg) mainly by microbial activity [58,59]. IHg and MeHg have different fates in the aquatic environment, since both can be assimilated by biota, but only MeHg is bioaccumulated in the aquatic trophic network [58]. MeHg in sediment and water can be taken up by phytoplankton and transferred to secondary consumers, such as fishes, invertebrates, and, consequently, birds [60,61]. Eisler [57] and Burger and Gochfeld [48] proposed 5.0 mg Kg^−1^ as the critical concentration of Hg in bird feathers associated with adverse effects. On the contrary, Goutner et al. [62] found no visible effects on the growth of Squacco Heron *Ardeola ralloides* when Hg reached 6.5 mg Kg^−1^, while Connell et al. [63] suspected adverse effects in herons, even below the threshold level for Hg.

Mercury can be excreted both in eggshells and feathers. Its storage in the latter, being one of the preferred ways of excretion, therefore, can indicate the effect of a physiological protective action of the organism against the metal [64].

The average Hg concentration found in the present study, is below the threshold level and ranges from 0.7 mg Kg^−1^ in Little Egret and Cattle Egret to 2.8 in Grey Heron; however some individuals show higher values (namely 2.3, 3.2, and 4.2 mg Kg^−1^ in Little Egret, Grey Heron, and Cattle Egret, respectively). Moreover, Hg levels are about two- to three-fold higher in adults than in chicks for all the species (Table 2). As reported above, differences in mercury concentration are attributable to diet. However, different bioaccumulation levels in adults can also be affected by other variables, such as age or molting that occurs far away from the reproductive site. In addition, Hg concentration in chick feathers reflects the presence of contaminated prey in foraging areas surrounding the colony, resulting in a good predictor of the local environmental pollution [65]. Differently, Wang [66] detected Hg values ten-fold higher in adult Grey Heron (0.55 ± 0.06 mg Kg^−1^) than in chicks (0.055 ± 0.024 mg Kg^−1^) from coastal China and lower than those we reported (Table 2), and confirmed that the biomagnification process was usually significantly reduced in chicks as compared with adults [21,67]. Consequently, the higher Hg levels detected in Grey Heron chicks examined in the present study suggested the need for further investigation, as already suggested by Burger and Gochfeld [16] and Connell et al. [63].

**Figure 4:**
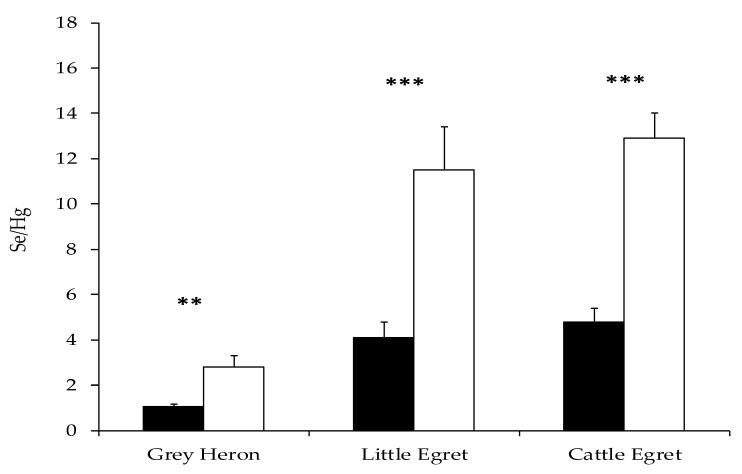
Se/Hg molar ratio and standard error in adults’ (black) and chicks’ (white) feathers. The asterisks mark a statistically significant difference at *p* < 0.01 (**) and *p* < 0.001 (***) (Mann–Whitney U test).

Selenium concentrations range from 1.18 mg Kg^−1^ in Cattle Egret to 1.45 mg Kg^−1^ in Grey Heron, with a maximum value of 2.15 mg Kg^−1^ in Little Egret and 1.98 mg Kg^−1^ in Grey Heron and Cattle Egret. Selenium (Se) is an essential element, however, it can cause behavioral abnormalities, reproductive deficits, and increased mortality at concentrations slightly higher than those needed [68,69]. High concentrations of Se in liver (19–130 mg Kg^−1^, [4]) and feathers (3.8–26 mg Kg^−1^, [70]) have increased embryos mortality. There is no threshold level, although Heinz [70] found related sublethal effects at 1.8 mg Kg^−1^ concentration. Nevertheless, Se in the diet offers protection against the harmful effects of Hg, as observed in numerous studies [16] carried out also on juveniles [71]. Specifically, the metabolic action occurred when the Se/Hg molar ratio was higher than a unit [72,73]. Interestingly, in the present investigation, the molar ratio Se/Hg is higher than a unit in juveniles of the three species and significantly lower in adults (Mann–Whitney U test, Gray Heron: z = −2.66, n = 17, *p* = 0.008; Little Egret: z = −3.49, n = 23, *p* < 0.0001; Cattle Egret: z = −3.79, n = 25, *p* < 0.0001, see Figure 4).

### 4.4. Trophic Level

In waterbirds, the trophic level of predator and prey may be a crucial parameter in Hg bioaccumulation and biomagnification [74]. As observed in two fish-eaters, Grey Heron and Great Egret *Egretta alba*, the difference in Hg concentration seems to be due to the trophic level of prey [66]. Indeed, Grey Heron feeds mainly on *Perccottus glehni* and *Misgurnus moloity*, while the herbivorous *Carassius auratus* is the main food of Great Egret. 

Hg concentrations (see Table 1 and Table 2) differ significantly among the investigated species, and they are significantly higher in adults than in juveniles, suggesting that heron diet could account for such a difference. In the heronries of NW Italy, the Cattle Egret is a generalist feeder, well adapted to all agricultural habitat; its dietary spectrum ranges from small fishes to arthropods, insects, and amphibians. On the contrary, medium-size fishes, reptiles, and small mammals are everyday items in the Grey Heron diet, while that of Little Egret relies on small fish, frogs, aquatic crustaceans, and tadpoles.

Since the late 1990s, the Little Egret diet has changed for several reasons, such as a general reduction of the watershed, different water use in agriculture, and, probably, climate changes. As a result, diet bulk has shifted from fish and amphibians to terrestrial or mud species preys, such as worms, Louisiana crayfish *Procambarus clarkii*, terrestrial arthropods, and *Gryllotalpa* sp. [75]. As previously stated by Alvarez et al. [76] and also the results of the present study, bird species with terrestrial feeding habits show Hg contaminations lower than waterbirds. The relevance of fish, which is the primary source of Hg in the diet of the three investigated species, is highlighted by the results shown in Figure 1. In particular, the closeness of Little Egret centroid to Cattle Egret indicates that the two species share similar feeding habitats, confirming the shift of the Little Egret diet towards a diet based on insect and arthropods. 

## 5. Conclusions

The results obtained in the present study confirm that most of the concentrations of the analyzed elements reflect the background concentration, and they are below the threshold levels, i.e., below concentration levels recognized as harmful for life, reproduction, and survival. On the contrary, Hg levels in some cases are close to the critical value, indicating that a potentially detrimental effect on individuals cannot be excluded and require in-depth investigation. Differently from adults, Hg levels in chicks are always lower and exclusively relate to the presence of contaminated prey in foraging areas surrounding the colony; Hg pattern in the three heron species confirms the dietary shift of the Little Egret during the last decade. Finally, the Se/Hg ratio is higher in chicks than in adults, and as reported in the literature, higher levels of Se may protect chicks against the adverse effects of Hg. Harmful and acute effects on the local heron population are unlikely; the conservation status of populations in northern Italy is probably more affected by other factors, such as climate change, altered aquatic environment, and, consequently, food quality.

## Figures and Tables

**Table 1:** Descriptive statistics (mean ± SE, minimum-maximum) for trace elements’ concentrations in feathers of Grey Heron (*Ardea cinerea*), Little Egret (*Egretta garzetta*) and Cattle Egret (*Bubulcus ibis*). Data are expressed as mg Kg^−1^. Tukey post hoc test shows significant differences at *p* < 0.05 (*), *p* < 0.01 (**), and *p* < 0.001 (***) for ab (*A.cinerea-E.garzetta*), bc (*E.garzetta-B.ibis*) and ac (*A.cinerea-B.ibis*) pairs; ns = not significant. ^♦^ Method quantification limit (MQL) = 0.05 (see Table A1 for MQLs values).

	a	b	c			Tukey Test		
	*A. cinerea* (n = 17)	*E. garzetta* (n = 23)	*B. ibis* (n = 25)	F_2,62_	*p*	ab	bc	ac
As	0.27 ± 0.03 (0.12–0.42)	0.6 ± 0.1 (0.1–1.9)	0.5 ± 0.1 (0.1–2.3)	4.47	0.015	*	ns	ns
Cd^♦^	0.04 ± 0.02 (< ^♦^MQL–0.06)	0.05 ± 0.01 (0.05–0.30)	0.06 ± 0.01 (0.05–0.19)	0.28	0.281	ns	ns	ns
Co	0.09 ± 0.08 (0.02–0.10)	0.07 ± 0.01 (0.02–0.15)	0.07 ± 0.01 (0.02–0.13)	2.78	0.069	ns	ns	ns
Cr	0.59 ± 0.03 (0.43–0.79)	0.48 ± 0.07 (0.08–1.81)	0.40 ± 0.04 (0.15–0.78)	3.04	0.051	ns	ns	*
Cu	10.3 ± 0.5 (7.1–11.9)	13.3 ± 0.8 (8.3–24.7)	17 ± 3 (5–80)	4.20	0.019	**	ns	ns
Fe	63 ± 9 (25–125)	62 ± 11 (6–248)	57 ± 5 (13–142)	0.14	0.872	ns	ns	ns
Hg	2.8 ± 0.2 (1.5–3.2)	0.7 ± 0.1 (0.2–2.3)	0.7 ± 0.2 (0.2–4.2)	57.48	<0.001	***	ns	***
Mn	13 ± 1 (8–18)	7 ± 1 (1–24)	7.6 ± 0.9 (2.3–23.2)	20.19	<0.001	***	ns	**
Ni	1.3 ± 0.2 (0.4–2.8)	0.57 ± 0.07 (0.11–1.58)	0.50 ± 0.05 (0.14–1.15)	12.29	<0.001	***	ns	***
Pb	0.67 ± 0.04 (0.45–0.84)	0.53 ± 0.06 (0.15–1.14)	0.64 ± 0.08 (0.15–1.67)	1.23	0.299	ns	ns	ns
Se	1.45 ± 0.09 (1.00–1.98)	1.22 ± 0.07 (0.73–2.15)	1.18 ± 0.08 (0.57–1.98)	5.71	0.005	ns	ns	*
Zn	69 ± 32 (50–83)	47 ± 4 (6–86)	45 ± 3 (9–64)	15.44	<0.001	***	ns	***

**Table 2:** Differences between trace elements’ concentrations in adults and juveniles feathers of Grey Heron (*A. cinerea*), Little Egret (*E. garzetta*), and Cattle Egret (*B. ibis*); ns = not significant. Mean ± SE. Data are expressed as mg kg^−1^.

	*A. cinerea*				*E. garzetta*				*B. ibis*			
	Ad. (n = 12)	Juv. (n = 5)	t	*p*	Ad. (n = 15)	Juv. (n = 8)	t	*p*	Ad. (n = 17)	Juv. (n = 8)	t	*p*
As	0.31 ±0.04	0.19 ± 0.03	2.51	0.021	0.7 ± 0.1	0.4 ± 0.1	1.25	ns	0.5 ± 0.1	0.6 ± 0.2	−0.27	ns
Cd	0.04 ±0.01	0.04 ±0.01	0.02	ns	0.06 ± 0.02	0.04 ± 0.01	1.02	ns	0.06 ± 0.01	0.06 ± 0.01	0.03	ns
Co	0.085 ±0.001	0.096 ± 0.002	−4.81	0.001	0.08 ± 0.01	0.052 ± 0.005	2.44	ns	0.069 ± 0.008	0.073 ± 0.008	0.10	ns
Cr	0.61 ± 0.04	0.53 ± 0.07	1.03	ns	0.6 ± 0.1	0.31 ± 0.06	2.30	0.032	0.39 ± 0.05	0.42 ± 0.06	−0.36	ns
Cu	11.2 ± 0.2	8.0 ± 0.9	4.71	0.001	15 ± 1	10.5 ± 0.6	2.80	0.011	15 ± 2	22 ± 8	−1.14	ns
Fe	45 ± 9	105 ± 12	−3.81	0.002	71 ± 17	45 ± 9	1.07	ns	54 ± 5	63 ± 13	−0.82	ns
Hg	3.20 ± 0.01	1.8 ± 0.3	9.19	0.001	0.9 ± 0.1	0.32 ± 0.02	3.22	0.004	0.9 ± 0.2	0.21 ± 0.01	2.97	0.009
Mn	15 ± 1	7.64 ± 0.04	5.16	0.001	8 ± 1	4.6 ± 0.7	1.59	ns	6.3 ± 0.8	10 ± 2	−2.37	0.027
Ni	1.4 ± 0.2	0.9 ± 0.5	0.98	ns	0.7 ± 0.1	0.33 ± 0.07	2.79	0.013	0.47 ± 0.05	0.6 ± 0.1	−1.04	ns
Pb	0.70 ± 0.05	0.61 ± 0.06	0.94	ns	0.62 ± 0.06	0.38 ± 0.08	2.26	0.035	0.7 ± 0.1	0.54 ± 0.09	0.85	ns
Se	1.32 ± 0.07	1.8 ± 0.2	2.83	0.013	1.15 ± 0.09	1.4 ± 0.1	1.45	ns	1.2 ± 0.1	1.08 ± 0.09	0.87	ns
Zn	65 ± 3	77 ± 7	−1.76	ns	45 ± 6	50 ± 5	0.56	ns	45 ± 3	46 ± 5	−0.11	ns

## Appendix A

**Table A1:** Detection and quantification limits of trace elements.

	IDL	IQL	MDL	MQL
	(µg L^−1^)	(µg L^−1^)	(mg Kg^−1^)	(mg Kg^−1^)
**As**	0.06	0.2	0.03	0.1
**Cd**	0.03	0.1	0.02	0.05
**Co**	0.01	0.03	0.01	0.02
**Cr**	0.05	0.15	0.03	0.1
**Cu**	0.01	0.03	0.01	0.02
**Fe**	0.6	1.8	0.3	0.9
**Hg**	0.06	0.2	0.03	0.1
**Mn**	0.01	0.03	0.01	0.02
**Ni**	0.01	0.03	0.01	0.02
**Pb**	0.01	0.03	0.01	0.02
**Se**	0.2	0.6	0.1	0.3
**Zn**	0.05	0.15	0.03	0.1
